# Maximization of brackish water productivity for the sustainable production of striped catfish (*Pangasianodon hypophthalmus*) and grain sorghum (*Sorghum bicolor* (L.) Moench) cultivated under an integrated aquaculture–agriculture system

**DOI:** 10.1007/s11356-024-33216-x

**Published:** 2024-04-19

**Authors:** Fahad Kimera, Muziri Mugwanya, Khaled Madkour, Mahmoud A. O. Dawood, Hani Sewilam

**Affiliations:** 1 Center for Applied Research On the Environment and Sustainability (CARES), School of Science and Engineering, The American University in Cairo, AUC Avenue, P.O. Box 74, New Cairo, 11835 Egypt; 2 Animal Production Department, Faculty of Agriculture, Kafrelsheikh University, Kafr El-Sheikh, 33516, Egypt; 3 Department of Engineering Hydrology, RWTH Aachen University, Aachen, 52062 Germany

**Keywords:** Brackish water, Salinity, Integrated aquaculture–agriculture system, Sorghum, Stripped catfish

## Abstract

Freshwater scarcity, salinity, and poor soil fertility are the major challenges affecting both food and feed productions in arid and semi-arid regions of the world. Utilization of brackish water in the production of saline-tolerant fish and valuable field crops under an integrated system is promising in the maximization of yield per crop. The aim of this study, therefore, was to (1) assess the effect of saline aquaculture wastewater on the growth, yield, forage quality, and nutritive composition of sorghum seeds and (2) assess the effect of different water qualities on the survival, growth performance, and health status of *Pangasianodon hypophthalmus*. The experiment was conducted in a randomized completely block design of four salinity treatments with three replicates, i.e., control (freshwater mixed with inorganic fertilizers), 5000 ppm, 10,000 ppm, and 15,000 ppm. Our results indicated that although the control exhibited the highest growth (plant height, leaf number, internode number, leaf area, and soil–plant analysis development), grain, and forage yield, no significant differences were noted among the treatments. Likewise, no significant difference in the grain nutrient composition was noted among all the treatments. Assessment of the forage quality revealed improved crude protein content in the control compared to the saline treatments. However, no significant differences in the leaves and stalks fiber fractions were noted among all the treatments. Furthermore, rumen fermentation in terms of in vitro digestibility indicated no significant differences in the in vitro digestible dry matter, digestible organic matter, metabolic energy, net energy, microbial protein, short-chain fatty acids, and total dissolved nutrients among the treatments. However, rearing *P. hypophthalmus* in water salinities exceeding 10,000 ppm reduced the growth performance and health status of fish. Therefore, the integration of sorghum and *P*. *hypophthalmus* production in water salinities not exceeding 5000 ppm is a viable alternative to maximize brackish water productivity in freshwater-scarce regions.

## Introduction

Climate change has altered the global weather and water patterns leading to severe droughts, water shortages, and increasing salinity in several regions of the world (Ismail and Go 2021). According to the United Nations Sustainable Development (UNSDG), half of the world’s population has already experienced severe water scarcity for at least one month in a year, and by 2030, it is expected that ~ 700 million people will be affected by severe water scarcity (Ismail and Go 2021). This is attributed to the rapid growth in human population coupled with the increasing water demand and usage which together will put more pressure on the available limited freshwater resources (Huang et al. 2012; Arnell 2018; Mishra et al. 2021; Kumar et al. 2021). For example, Egypt, one of the most populous countries in Africa, is currently facing severe water shortages and her water poverty threshold has reached less than 500 m^3^ of water per capita per year (Rashid 2012). Moreover, the recent filling and operation of the Grand Ethiopian Renaissance Dam have further threatened Egypt’s freshwater supply, food security, and economic development. In the same regard, the over-dependence of the country’s agricultural sector on synthetic fertilizers and underground water for irrigation of water-intensive crops such as wheat, corn, and rice has further lowered crop yields due to the increasing soil salinity and land degradation (Awaad et al. 2010; Abd El-Gawad and Morsy 2017; Ding et al. 2020; Moghazy and Kaluarachchi 2020). To overcome the aforementioned challenges, there is a need for the implementation of realistic and scientifically feasible interventions to safeguard the country’s water and food security.

Maximization of water productivity through the introduction of salt and drought-tolerant crops to the Egyptian cropping patterns will aid in tackling crop yield losses due to the increasing soil salinity and freshwater scarcity (El Demerdash et al. 2022). Moreover, the integration of salt-tolerant crops with aquaculture (i.e., Integrated Aqua-Agriculture Systems [IAAS]) will promote more crop (i.e., plant and fish) yield per drop thus contributing to double economic benefits. IAAS operates on a principle of wastewater reuse where aquaculture effluents are utilized as both irrigation water and nutrient sources for plants (Farrag et al. 2021; Sewilam et al. 2022). Previous studies have shown significant improvements in crop yield as well as crop water and nutrient use efficiency in plants cultivated under IAAS (Dey et al. 2010; Mariscal-Lagarda et al. 2012; M.Moursy et al. 2019; Farrag et al. 2021; Sewilam et al. 2022). Likewise, several studies have also shown improved water quality, survival, and growth performance of fish reared under IAAS (Mariscal-Lagarda et al. 2012; Farrag et al. 2021; Sewilam et al. 2022). For successful implementation and operation of the IAAS especially under saline conditions, the selection of suitable fish and crops of high economic value is paramount.

Stripped catfish (*Pangasianodon hypophthalmus*) is a highly tolerant freshwater fish to various salinity ranges and thus can be considered a potential candidate species for inland saline aquaculture (Kumar et al. 2017; Hossain et al. 2021). Its high market value is attributed to its palatability, high protein content, lower number of spines, rapid growth rate, easy acceptance of artificial feed, and ability to tolerate environmental stress (high temperature, crowding, and salinity) (Ahmed et al. 2010; Singh and Lakra 2012; Kumar et al. 2017; Jahan et al. 2019). Previous studies have shown that rearing *P*. *hypophthalmus* at high water salinities reaching 10,000 to 15,000 ppm does not negatively impact the growth performance and the general well-being of fish (Nguyen et al. 2014; Kumar et al. 2017; Hieu et al. 2021). Likewise, previous studies have also shown that *P*. *hypophthalmus* can tolerate crowding stress (Nageswari et al. 2022b, a), high-temperature stress (28–32 °C) (Shahjahan et al. 2018; Islam et al. 2019), and poor water quality (Okomoda et al. 2019; Hasibuan et al. 2021). However, *P*. *hypophthalmus* wastewater (effluents) is highly rich in nutrients and its poor disposal could lead to environmental pollution (De Silva et al. 2010; Anka et al. 2014; Nhut et al. 2019; Meena et al. 2022). Therefore, the utilization of *P*. *hypophthalmus* wastewater as a water and nutrient source for plants could hinder environmental pollution and improve plant growth and yields.

Sorghum (*Sorghum bicolor* (L.) Moench) is one of the most cultivated foods and fodder crops in Africa with the potential to improve food and feed security in drought-stricken, water-scarce, and saline regions (Bavei et al. 2011; Hadebe et al. 2017; Mansour et al. 2021; Amombo et al. 2022). This is attributed to its drought tolerance, moderate tolerance to salinity, as well as the ability to grow in poor nutrient soils (Hufnagel et al. 2014; Mansour et al. 2021; Abreha et al. 2022). Previous studies have shown that cultivation of sorghum under moderately saline conditions does not significantly impact its growth, grain or forage yield, and forage quality (Francois et al. 1984; Hedayati-Firoozabadi et al. 2020; Gois et al. 2022). To the best of our knowledge, no studies have so far been conducted on the integration of *P*. *hypophthalmus* aquaculture and *S. bicolor* production in IAAS under brackish water conditions. The current study, therefore, aims to (1) assess the effect of saline aquaculture wastewater on the growth, yield, forage quality, and nutritive composition of *S. bicolor* grains under an IAAS and (2) assess the effect of different water qualities on fish survival, growth performance, and health status.

## Material and methods

### Plant material and experimental design

**Fig. 1:**
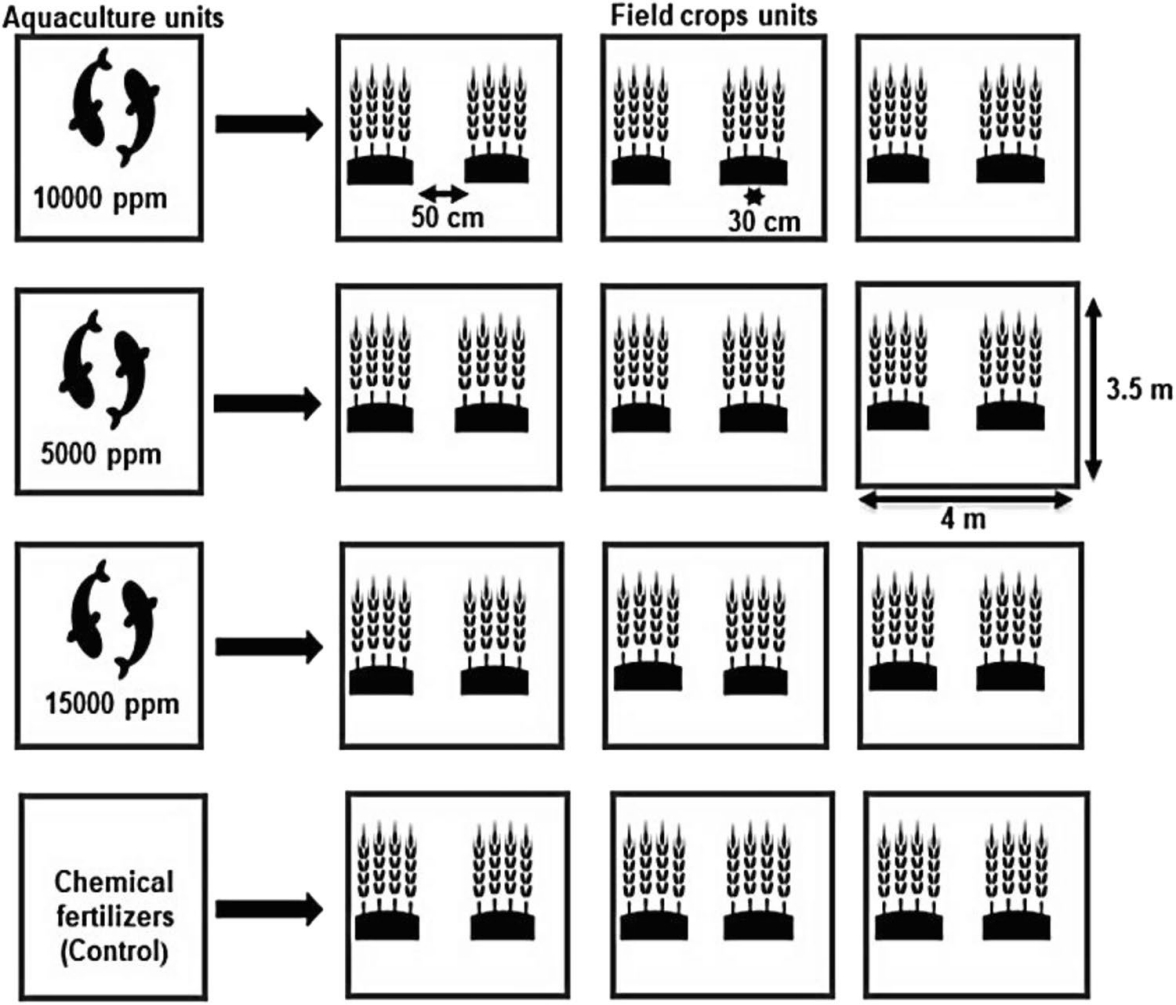
Schematic diagram showing the experimental layout

Sorghum seeds were obtained from the Agricultural Research Center (ARC) in Giza, Egypt. A field experiment was conducted between August and November 2021 at the Center for Applied Research on the Environment and Sustainability (CARES), the American University in Egypt, New Cairo, Egypt (30° 01′ 11.7″ N31° 29′ 59.8″ E). The experiment was conducted in a randomized completely block design of four salinity treatments with three replicates, i.e., control (freshwater mixed with inorganic fertilizers), 5000 ppm, 10,000 ppm, and 15,000 ppm (Fig. 1). Table 1 shows the chemical properties of the salt used in this study (Mugwanya et al. 2023). The soil's physical and chemical properties are presented in Table 2. Within the growing season, the maximum average temperature was 27.5 °C, average relative humidity was 62%, solar radiation was 207.58 Wm^2^, and wind speed was 1.56 m s^−1^.

**Table 1:** Chemical properties of the salt used in this study

Sodium chloride	98.50%	Bicarbonate	4 × 10^−3^%
Soluble matter	0.57%	Copper	2 × 10^−6^%
Insoluble matter	0.02%	Iron	3 × 10^−6^%
Moisture content	0.23%	Potassium iodide	5.3 × 10^−3^%
Magnesium	0.07%	Mercury	5 × 10^−6^%
Calcium	0.07%	Lead	2 × 10^−5^%
Potassium	0.02%	Arsenate	2 × 10^−5^%
Sulfate	0.31%	Cadmium	8 × 10^−7^%

**Table 2:** Physical and chemical properties of the soil used in the study

			Anions				Cations			
pH	EC (ppm)	SP	CO_3_^_^ (meq L^−1^)	HCO_3_^_^ (meq L^−1^)	Cl^−^ (meq L^−1^)	SO_4_^_^ (meq L^−1^)	Ca^++^ (meq L^−1^)	Mg^++^ (meq L^−1^)	Na^+^ (meq L^−1^)	K^+^ (meq L^−1^)
7.6	1376	23.00	-	2.36	8.47	11.61	10.61	6.28	5.13	0.43
			Available macro- and micronutrients (mg kg^−1^)							
			N	P	K	Mn	Zn	Fe	Cu	
			47.00	14.54	48.00	0.43	0.16	1.44	0.06	

*EC* electroconductivity, *SP* saturation point, *CO*_*3*_^*_*^ carbonate, *HCO*_*3*_^*_*^ hydrogen carbonate, *Cl*^*−*^ chloride, *SO*_*4*_^*_*^ sulfate, *Ca*^++^ calcium ions, *Mg*^++^ magnesium ions, *Na*^+^ sodium ions, *K*^+^ potassium ions, *N* nitrogen, *P* phosphorus, *K* potassium, *Mn* manganese, *Zn* zinc, *Fe* iron, *Cu* copper

### Crop cultivation

Seeds were hand-sewn in rows with inter and intra-row spacing of 30 cm and 50 cm respectively based on a planting density of 66,667 plants ha^−1^. Weeding was done by hand and plants were drip irrigated according to crop water requirements. The crop water requirements were estimated using the Food and Agriculture Organization (FAO)-CROPWAT model (Gabr 2022). As such, water was pumped from the tanks (i.e., aquaculture units and the control) to the designated grow beds by an automated drip irrigation system to irrigate the plants according to the set conditions. Pest and insect control was conducted according to the recommendations of the Egyptian Ministry of Agriculture. Likewise, fertigation (control treatment) for sorghum was performed according to the recommendations of the Egyptian Ministry of Agriculture.

### Sampling and agronomical trait measurements

Six plants within the borders per replicate were randomly tagged for agronomical trait measurements. At each data collection time point, plant heights were measured from the crown to the terminal growing point of the plant using a meter rule. Stalk diameters were measured from the second internode from the bottom-up of the plant using digital vernier calipers and averages were determined. The number of internodes and leaf number per plant was obtained by counting and averages were determined. The leaf area was calculated as shown below according to the equation by Elsahookie and Cheyed (2014).

$$\mathrm{Leaf \ area}=L\times W\times 0.75$$

where *L* and *W* are the leaf length and leaf width, respectively.

Chlorophyll content was measured in the early morning at each data collection time point using an MC-100 chlorophyll meter from Apogee Instruments, Inc., Utah, USA, and data was expressed as soil–plant analysis development (SPAD) averages.

For the determination of fresh weights, six plants per replicate were divided into stalks, leaves, and panicles. Stalks were cut into smaller pieces of approximately 5 cm. All plant fractions were weighed to obtain the fresh weights and then oven-dried at a constant weight at 70 °C for 3 days to obtain the dry weights, and the data were expressed as grams per plant.

### Seed nutrient composition analysis

For each treatment, a 300-mg sample of seeds was obtained for microwave digestion (Model: speed wave Entry DAP-60 K). Briefly, the seeds were placed in a digestion vessel and 65% 3.0 ml of nitric acid (HNO_3_) and 35% hydrogen peroxide (H_2_O_2_) were added. The mixture was carefully stirred using a clean glass rod and gently shaken. The vessel was closed 10 min after shaking and the sample was heated in the microwave. After cooling, the clear solution was obtained and used for elemental analysis (phosphorus, calcium, magnesium, zinc, copper, manganese, and iron) using inductively coupled plasma–optical emission spectroscopy (ICP-OES, Model: Ultima 2 JY Plasma). All measurements were performed using an Agilent 4210 MP-AES fitted with a double-pass cyclonic spray chamber and a OneNeb Series 2 nebulizer. Nitrogen was supplied using an Agilent 4107 Nitrogen Generator. All wavelengths were selected from the MP Expert software library, according to the sensitivity that was required.

### Fiber fraction, nutrient composition, and in vitro digestibility of leaves and stalks

Fiber fraction analysis is a vital technique used for the determination of the forage quality of animal feeds. It takes into account the following parameters: neutral detergent fiber (NDF), acid detergent fiber (ADF), and acid detergent lignin (ADL). In this study, samples per replicate in each main treatment were pooled and taken to the Regional Center for Food and Feed, Giza, Egypt. Briefly, stalks and leaves were separately ground using a Wiley mill and the fine powder passed through a 1-mm screen. Neutral detergent fiber (NDF, AOAC no. 2002.04), acid detergent fiber (ADF, AOAC no. 973.18), and acid detergent lignin (ADL, AOAC no. 973.18) were sequentially determined by semiautomatic ANKOM220 Fiber Analyzer (ANKOM Technology, Macedon, NY, USA). Cellulose (ADF-ADL), hemicellulose (NDF-ADF), and lignin were calculated from the organic matter of the detergent fiber fractions respectively. The total nitrogen content of the samples was determined by the Kjeldahl technique followed by the determination of concentrations of crude protein (CP) according to the Association of Official Analytical Chemists 2016 (AOAC no.984.13 and no. 968.06, respectively). Fat and fiber contents were determined according to the approved methods given in AOAC (1984).

Assessment of the quality of animal feeds has for a long time been performed by in vitro digestibility and gas production techniques. This technique involves the use of Rumen fluid extracted from animals as an inoculum to mimic the in vivo fermentation of feed, thus allowing a proper estimation of the nutritive composition and fermentation kinetics of ruminant feeds through gas production. Merits of this technique over the in vivo fermentation in the determination of the nutritive value of feedstuffs include the following: It is cheaper, faster, less labor intensive, and suitable for both small quantities of feed and large-scale evaluation of ruminant feeds (Getachew et al. 1999). In this study, the gas production technique was performed according to Menke and Steingass (1988) at the Regional Center for Food and Feed, Agricultural Research Center, Giza. Briefly, ammonium-free rumen fluid was collected in equal proportions from two animal donors (sheep) before their morning feed and put into thermo flasks. The rumen fluid was later filtered through a 1-mm sieve and the obtained filtrate was incubated at 39 °C. Rumen 27 liquor and buffer solution were mixed in a ratio of 1:2 (v/v), and all laboratory procedures for handling rumen liquor were conducted under a continuous flow of carbon dioxide gas. Two hundred-milligram test samples were fed into 100-ml capacity graduated plastic syringes, and the lubricated pistons were inserted into the syringes. Thirty milliliters of rumen liquor (inoculum) was introduced into the plastic syringes via silicon tubes at the tips of the syringes, and these were subjected to incubation (± 39 °C). Gas production was measured at 2, 4, 6, 8, 10, 12, 14, 16, 18, 20, 22, and 24 h. This experiment was conducted in triplicates. In vitro true digestible dry matter (IVTD), digestible organic matter (DOM), metabolic energy (ME), and net energy (NE) were calculated as described by Menke and Steingass (1988). Total digestible nutrients (TDN) were calculated from ME values as per the equation of NRC (1989). Microbial protein (MP) was calculated as described by Czerkawski (1986), whereas short-chain fatty acids (SCFA) were calculated as described by Getachew et al. (1999).

### Fish growth performance, hematological, and serum biochemical parameters

Stripped catfish (*Pangasianodon hypophthalmus*, initial weight 6.0 g) were obtained from a local distributor and stocked in each tank at a stocking density of 100 fish/900 l. The fish were fed two to three times daily with commercial pellets supplied by Skretting Egypt. The pellets contained 28% crude protein, 5% crude lipid, 6% crude fiber, 13% ash, and 9% moisture. The feeding pattern and frequency were according to the fish biomass percentage of 2–3% depending on the growth and satiation. Fish growth parameters such as feed conversion ratio (FCR), specific growth rate (SGR), feed intake (FI), body weight gain (BWG), survival rate (SR%), and condition factor (CF) were calculated according to the formula below.

$$\begin{array}{l}\mathrm{BWG}=\mathrm F\mathrm i\mathrm n\mathrm a\mathrm l\;\mathrm b\mathrm o\mathrm d\mathrm y\;\mathrm w\mathrm e\mathrm i\mathrm g\mathrm h\mathrm t-\mathrm I\mathrm n\mathrm i\mathrm t\mathrm i\mathrm a\mathrm l\;\mathrm b\mathrm o\mathrm d\mathrm y\;\mathrm w\mathrm e\mathrm i\mathrm g\mathrm h\mathrm t\\\mathrm{FCR}=\mathrm{FI}/\mathrm{BWG}\\\begin{array}{l}\mathrm{SGR}=(\ln\left(\mathrm F\mathrm i\mathrm n\mathrm a\mathrm l\;\mathrm b\mathrm o\mathrm d\mathrm y\;\mathrm w\mathrm e\mathrm i\mathrm g\mathrm h\mathrm t\right)-\ln(\mathrm I\mathrm n\mathrm i\mathrm t\mathrm i\mathrm a\mathrm l\;\mathrm b\mathrm o\mathrm d\mathrm y\;\mathrm w\mathrm e\mathrm i\mathrm g\mathrm h\mathrm t))/\mathrm N\mathrm u\mathrm m\mathrm b\mathrm e\mathrm r\;\mathrm o\mathrm f\;\mathrm d\mathrm a\mathrm y\mathrm s\\\begin{array}{l}\mathrm{CF}=(\mathrm F\mathrm i\mathrm n\mathrm a\mathrm l\;\mathrm b\mathrm o\mathrm d\mathrm y\;\mathrm w\mathrm e\mathrm i\mathrm g\mathrm h\mathrm t\;\left(\mathrm g\right)/\left(\mathrm{fish}\;\mathrm{body}\;\mathrm{length}\right)^3\;\left(\mathrm{cm}\right)^3\times100\\\text{SR}=(\mathrm N\mathrm u\mathrm m\mathrm b\mathrm e\mathrm r\;\mathrm o\mathrm f\;\mathrm f\mathrm i\mathrm s\mathrm h\;\mathrm a\mathrm t\;\mathrm t\mathrm h\mathrm e\;\mathrm e\mathrm n\mathrm d\;\mathrm o\mathrm f\;\mathrm t\mathrm h\mathrm e\;\mathrm s\mathrm t\mathrm u\mathrm d\mathrm y/\mathrm N\mathrm u\mathrm m\mathrm b\mathrm e\mathrm r\;\mathrm o\mathrm f\;\mathrm f\mathrm i\mathrm s\mathrm h\;\mathrm a\mathrm t\;\mathrm t\mathrm h\mathrm e\;\mathrm b\mathrm e\mathrm g\mathrm i\mathrm n\mathrm n\mathrm i\mathrm n\mathrm g\;\mathrm o\mathrm f\;\mathrm t\mathrm h\mathrm e\;\mathrm e\mathrm x\mathrm p\mathrm e\mathrm r\mathrm i\mathrm m\mathrm e\mathrm n\mathrm t)\times100\end{array}\end{array}\end{array}$$

For hematological parameters, a group of three fish per treatment was randomly collected and a 1-ml sterile syringe was used to draw blood from the mid-ventral line behind the anal fin and collected in purple top EDTA blood collection tubes and immediately sent to the lab for complete blood count (CBC) test. Of blood per treatment, 150 µl was used for determining the CBC using the human automatic hematology analyzer (XP-300, Sysmex Corporation).

Serum was obtained by centrifuging blood at 1000 × g for 3 min and used for the analysis of serum biochemical parameters. Briefly, 1 ml of serum was obtained and used for quantification of total protein, albumin, blood urea nitrogen (BUN), and creatinine using the Total Protein Biuret Reagent kit (Cat. No. 310 001), Albumin-BCG kit (Cat. No. 211 001), UREA/BUN-Liquizyme kit (Cat No. 318 002), and Creatinine-Jaffe kit (Cat. No. 234 001) from SPECTRUM, respectively. Globulin was calculated as Total protein − Albumin. Absorbance at different wavelengths was read using the BIOMED DIAGNOSTICS (Model: CLINI-CHEM) serum analyzer.

### Fish wastewater quality

Fish wastewater quality parameters such as water temperature, pH, and dissolved oxygen (DO) were closely monitored using automated digital Nilebot technologies by Conative Labs to fit the ideal required levels for catfish. Likewise, water quality parameters such as ammonia, ammonium, ammonia–nitrogen, nitrite–nitrogen, and nitrate–nitrogen were quantified once every 2 weeks until the end of the growing season using water analysis chemical kits from Hanna instruments. Briefly, 100 ml of water was collected from the tanks before feeding and immediately taken to the lab for analysis of ammonia, nitrite, and nitrate concentrations using a photometer along with the ammonia reagent kit (H193715-01), nitrite reagent kit (H193707-01), and nitrate reagent kit (H193728-01) from HANNA instruments. Specific absorbance of the nitrogenous elements was measured using the Aquaculture Photometer device (H183303). The device was set to display the concentrations of ammonia–nitrogen, ammonium, nitrate–nitrogen, and nitrite–nitrogen in milligrams per liter. Real-time analysis results were presented in the manuscript as mean values read from the photometer.

### Statistical analysis

Data sets were tested for normality before analysis of variance (ANOVA) was conducted. SPSS software (version 22) was used to carry out both one-way and two-way ANOVAs to test for significant differences (*p* < 0.05) between the treatments. Tukey’s HSD (honestly significant difference) test was used to compare differences among the treatment means when significant *F* values were observed at *p* < 0.05 level. Principal component analysis summarizing the variations in micro- and macronutrient compositions of sorghum seeds as well as correlograms showing correlation analysis of several parameters were constructed using the “*prcomp*” function and *corrgram* package of R Statistical Programming Language (version 4.1.0), respectively.

## Results

### Agronomical trait parameters

Table 3 summarizes data on the agronomical trait parameters of sorghum at different data collection time points. Results on plant height indicated no significant differences among all salinity treatments and the control at 30, 60, and 90 days after sowing (DAS). Plant heights increased with increasing plant maturity with 60 and 90 DAS significantly recording higher plant heights compared to 30 DAS. No significant interaction was noted between DAS × plant heights.

**Table 3:** Agronomical trait parameters of *Sorghum bicolor* at different data collection time points (30, 60, and 90 days after sowing)

Treatments	Plant height (cm)	Stalk diameter (mm)	Internode number plant^−1^	Leaf number plant^−1^	Leaf area (cm^2^)	SPAD
30 DAS						
Control	77.50^aB^ ± 15.38	16.29^aB^ ± 5.06	3.17^bC^ ± 0.86	7.67^aB^ ± 0.97	133.72^bB^ ± 31.24	325.01^aA^ ± 89.66
5000 ppm	72.32^aB^ ± 9.16	14.49^aB^ ± 3.92	2.22^aC^ ± 0.43	7.06^aB^ ± 1.26	139.61^abcB^ ± 30.53	307.51^aA^ ± 64.62
10,000 ppm	79.56^aB^ ± 6.22	15.21^aB^ ± 2.68	2.83^bC^ ± 0.51	6.83^aB^ ± 0.71	158.25^aB^ ± 45.28	326.75^aA^ ± 43.39
15,000 ppm	78.13^aB^ ± 12.79	15.36^aB^ ± 3.06	2.83^bC^ ± 0.58	7.00^aB^ ± 0.60	153.04^abB^ ± 49.16	319.33^aA^ ± 69.63
60 DAS						
Control	130.5^aA^ ± 19.27	23.80^aA^ ± 6.60	8.17^aB^ ± 2.59	10.17^aA^ ± 2.50	162.74^aA^ ± 38.22	264.04^aB^ ± 111.69
5000 ppm	126.13^aA^ ± 14.36	23.74^aA^ ± 7.38	5.78^bB^ ± 1.77	9.78^aA^ ± 2.56	197.88^aA^ ± 55.79	220.68^aB^ ± 88.59
10,000 ppm	128.11^aA^ ± 14.43	24.88^aA^ ± 5.62	6.06^bB^ ± 2.07	9.83^aA^ ± 2.23	178.68^aA^ ± 41.43	261.30^aB^ ± 97.36
15,000 ppm	125.66^aA^ ± 24.78	25.93^aA^ ± 4.98	5.92^bB^ ± 1.88	9.83^aA^ ± 2.56	186.35^aA^ ± 292.68	209.50^aB^ ± 94.74
90 DAS						
Control	133.67^aA^ ± 19.26	25.70^aA^ ± 6.64	13.33^aA^ ± 2.14	12.78^aB^ ± 3.06	115.10^aC^ ± 29.00	243.37^aB^ ± 112.04
5000 ppm	129.23^aA^ ± 14.43	25.50^aA^ ± 7.30	11.83^bA^ ± 2.07	6.67^bB^ ± 2.47	114.78^aC^ ± 36.24	197.68^aB^ ± 89.41
10,000 ppm	131.14^aA^ ± 14.39	26.68^aA^ ± 5.62	11.78^bA^ ± 1.73	5.67^bB^ ± 2.99	110.11^aC^ ± 34.11	239.30^aB^ ± 96.96
15,000 ppm	128.71^aA^ ± 24.84	27.93^aA^ ± 4.96	11.50^bA^ ± 2.07	5.67^bB^ ± 3.37	102.69^aC^ ± 25.55	189.00^aB^ ± 94.61

Data is expressed as mean ± SD (*n* = 6). Different lower superscript letters within each column indicate a significant difference within treatments (*p* < 0.05). Different upper superscript letters within each column indicate a significant difference on different days after sowing (DAS) (*p* < 0.05)

For stalk diameter, no significant differences were noted among all the salinity treatments and the control. However, stalk diameter increased with increasing plant maturity with 60 and 90 DAS significantly recording higher values for stalk diameter compared to 30 DAS. No significant interaction was noted between DAS × stalk diameter.

Results on the number of internodes showed that the control significantly recorded higher values for the number of internodes per plant compared to 5000, 10,000, and 15,000 ppm at 60 and 90 DAS. Overall, 90 DAS significantly recorded the highest values for the number of internodes per plant followed by 60 and 30 DAS, respectively. A significant interaction between DAS × number of internodes was noted (*p* < 0.05).

Results of leaf number indicated no significant differences in the average leaf number per plant among all salinity treatments and the control at 30 and 60 DAS. However, the average leaf number per plant of the control significantly increased (*p* < 0.05) by 39.98% compared to 5000, 10,000, and 15,000 ppm salinity treatments at 90 DAS. Overall, 60 DAS significantly recorded higher values for leaf number per plant compared to 30 and 90 DAS. A significant interaction was noted between DAS × leaf number per plant (*p* < 0.0001). For leaf area, no significant differences in the average leaf area were noted among the salinity treatments and the control at 60 and 90 DAS. Overall, 60 DAS significantly recorded higher values for the average leaf area compared to 30 and 90 DAS. No significant interaction was noted between DAS × leaf area.

Data on the leaf chlorophyll content (SPAD) indicated no significant differences among all salinity treatments and the control across all the data collection time points. However, 30 DAS significantly recorded higher SPAD values compared to 60 and 90 DAS. No significant interaction was noted between DAS × SPAD.

### Forage and grain yield

Results of the forage and grain yield are presented in Table 4. The control treatment recorded the highest forage fresh and dry yield compared to other treatments. However, no significant differences in yield were noted among all the treatments. Likewise, no significant differences in grain yield were noted among all the treatments.

**Table 4:** Results of forage and grain yield of sorghum at harvest

Treatments	Forage fresh yield (t ha^−1^)	Forage dry yield (t ha^−1^)	Grain yield (t ha^−1^)
Control	81.67^a^ ± 37.93	39.57^a^ ± 18.82	106.37^a^ ± 38.46
5000 ppm	62.02^a^ ± 29.79	31.87^a^ ± 14.40	77.61^a^ ± 10.66
10,000 ppm	54.48^a^ ± 37.23	29.53^a^ ± 15.67	72.36^a^ ± 10.29
15,000 ppm	50.17^a^ ± 28.40	28.14^a^ ± 16.61	58.36^a^ ± 19.73

Data is expressed as mean ± SD (*n* = 6). Different lower superscript letters within each column indicate a significant difference within treatments (*p* < 0.05)

### Correlation between yield and the average plant growth parameters of *Sorghum bicolor* cultivated under different salinity treatments

**Fig. 2:**
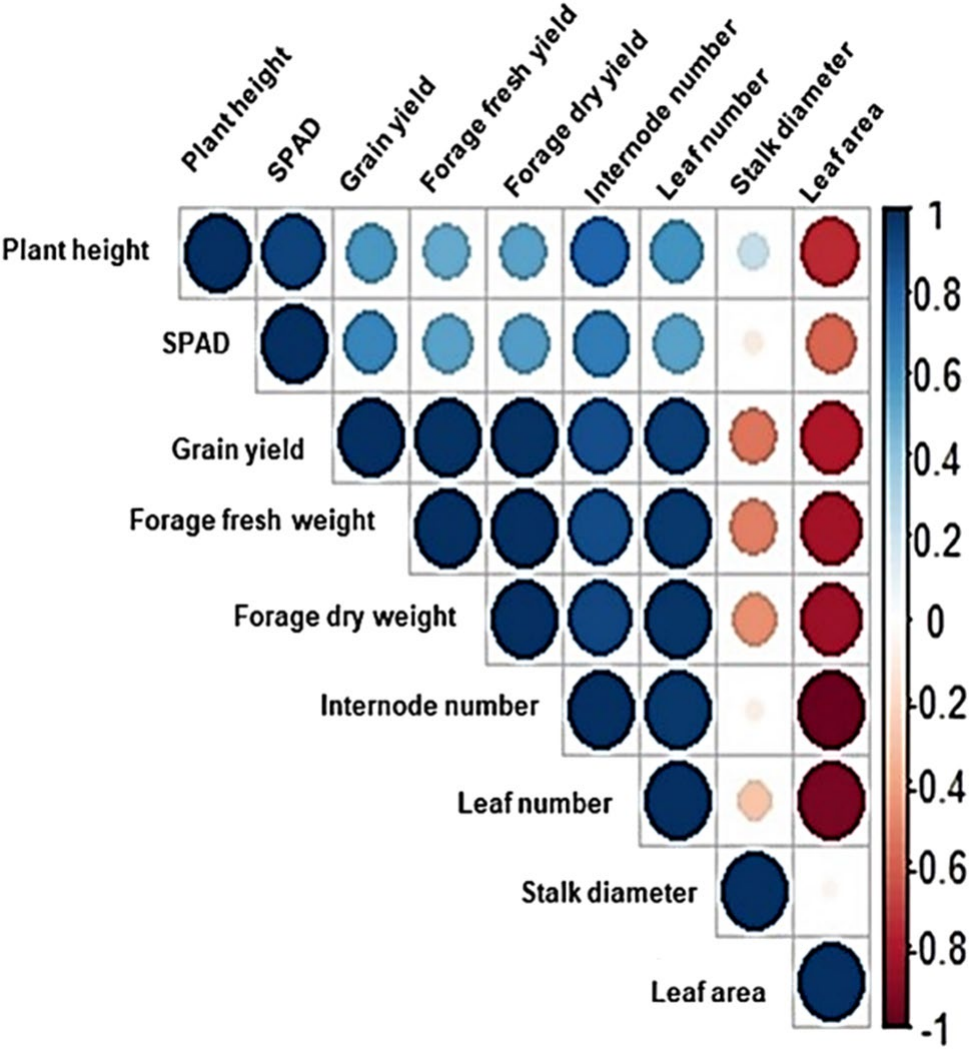
A correlogram showing correlation analysis of yield and the average growth parameters of sorghum. Blue and brown colors are positive and negative significant correlations, respectively, according to Pearson’s correlation analysis. The color intensity and circle size are proportional to the correlation coefficient

Figure 2 shows the results of the Pearson’s correlation analysis between yield and the average growth parameters of sorghum cultivated under different salinity treatments in the IAAS. A strong correlation was observed between the grain yield, forage fresh yield, forage dry yield, internode number, and leaf number. Plant heights were shown to be moderately correlated to SPAD, internode number, leaf number, grain yield, forage fresh yield, and forage dry yield. Likewise, SPAD was shown to be moderately correlated to grain yield, forage fresh yield, forage dry yield, internode number, and leaf number. Stalk diameter and leaf area were shown to be negatively correlated to all other plant growth parameters and yield.

### Seed nutrient composition and principal component and correlation analysis on nutrient composition of sorghum seeds

The results of the seed nutrient composition analysis are presented in Table 5. Data indicate no significant differences in the nutrient composition of macro elements (phosphorus, calcium, and magnesium) among all salinity treatments and the control. For microelement composition, 15,000 ppm had the highest zinc, copper, manganese, and iron content compared to other salinity treatments and the control, but no significant differences were noted.

**Table 5:** Results of seed nutrient composition of sorghum seeds under different treatments

Treatments	P (mg g^−1^)	Ca (mg g^−1^)	Mg (mg g^−1^)	Zn (mg g^−1^)	Cu (mg g^−1^)	Mn (mg g^−1^)	Fe (mg g^−1^)
Control	0.47^a^ ± 0.03	160.65^a^ ± 38.88	317.57^a^ ± 5.74	32.89^a^ ± 7.34	3.20^a^ ± 0.80	16.11^a^ ± 2.02	25.50^a^ ± 4.08
5000 ppm	0.40^a^ ± 0.11	130.35^a^ ± 24.55	307.58^a^ ± 17.63	25.46^a^ ± 2.62	2.57^a^ ± 0.04	12.99^a^ ± 1.87	26.23^a^ ± 2.36
10,000 ppm	0.47^a^ ± 0.02	139.61^a^ ± 39.87	319.43^a^ ± 38.64	28.58^a^ ± 2.55	3.62^a^ ± 0.58	15.02^a^ ± 4.22	34.24^a^ ± 8.60
15,000 ppm	0.46^a^ ± 0.02	185.86^a^ ± 37.45	313.09^a^ ± 11.04	34.80^a^ ± 5.13	4.17^a^ ± 1.89	24.94^a^ ± 5.33	40.01^a^ ± 10.65

Data is expressed as mean ± SD (*n* = 3). Different lower superscript letters within each column indicate a significant difference within treatments (*p* < 0.05)

Elements: *P* phosphorus, *Ca* calcium, *Mg* magnesium, *Zn* zinc*, Cu* copper, *Mn* manganese, *Fe* iron

**Fig. 3:**
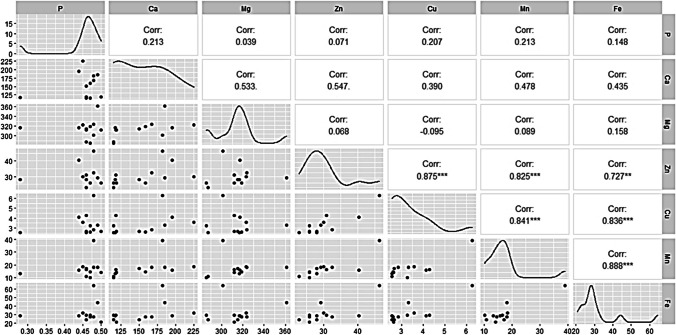
Correlation matrix for micro and macronutrient composition of sorghum grains. Statistical significance at p 0.05, 0.01, and 0.001 are shown using single, double, and triple asterisks respectively. Cu, copper; Mn, manganese; Zn, zinc; Fe, iron; P, phosphorus; Ca, calcium; Mg, magnesium

Results of the correlation matrix analysis (Fig. 3) showed a strong and positive correlation between copper (Cu) and zinc (Zn) (0.875), manganese (Mn) and Zn (0.825), as well as iron (Fe) and zinc (0.727). Likewise, a strong and positive correlation was noted between Mn and Cu (0.841), Fe and Cu (0.836), as well as Mn and Fe (0.888). Overall, macronutrients especially calcium (Ca) and magnesium (Mg) showed a moderate and positive correlation with micronutrients.

**Fig. 4:**
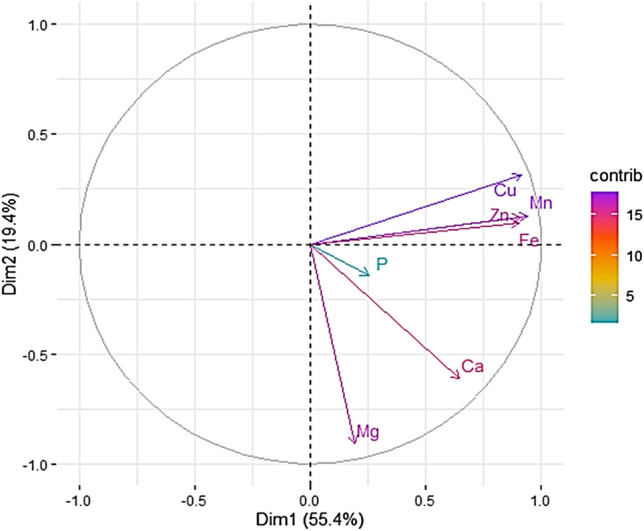
Principle component analysis (PCA) showing the correlations and variations in macro and micronutrient composition of sorghum grains. Cu, copper; Mn, manganese; Zn, zinc; Fe, iron; P, phosphorus; Ca, calcium; Mg, magnesium. Dim1 is PCA 1 (55.4%), and Dim2 is PCA 2 (19.4%)

Figure 4 summarizes the results of the principal component analysis. Phosphorus contributed less to the variation of the principal components (i.e., Dim 1 and Dim 2), whereas other elements contributed more to the variation of the principal components.

### Fiber fraction and nutrient composition of leaves and stalks

Table 6 summarizes the results on fiber fraction and nutrient composition of leaves and stalks. Fiber fraction analysis indicated no significant difference in the neutral detergent fiber (NDF), acid detergent fiber (ADF), and acid detergent lignin (ADL) of leaves and stalks among all the treatments. Likewise, no significant differences were noted in hemicellulose (HEM), cellulose (CEL), and lignin (LIG) content of leaves and stalks among all the treatments. The crude protein (CP) content in the leaves of the control treatment was significantly higher (*p* < 0.05) compared to that of other treatments. No significant differences in the leaves fat and fiber content were noted among all the treatments. For stalks, no significant differences were noted in the protein and fat content among all the treatments. However, the salinity treatment of 10,000 ppm significantly had a higher fiber content (*p* < 0.05) in stalks compared to the control. Overall, the cellulose content of leaves was lower (20–28%) compared to that of the stalks (30–32%). However, the CP of leaves was higher (5–9%) compared to that of stalks (2–3%).

**Table 6:** Fiber fraction and nutrient composition of leaves and stalks of *Sorghum bicolor* (L.) Moench cultivated under different salinity treatments

Treatment	NDF (%)	ADF (%)	ADL (%)	HEM (%)	CEL (%)	LIG (%)	CP (%)	Fats (%)	Fiber (%)
Leaf fiber fraction and nutrient composition									
Control	51.58^a^ ± 9.50	30.07^a^ ± 5.06	5.87^a^ ± 0.22	21.48^a^ ± 4.51	24.23^a^ ± 5.01	5.31^a^ ± 2.79	9.93^a^ ± 1.37	2.85^a^ ± 0.10	21.62^a^ ± 5.36
5000 ppm	58.15^a^ ± 2.03	34.12^a^ ± 0.56	5.03^a^ ± 0.23	18.19^a^ ± 9.89	20.57^a^ ± 4.99	10.71^a^ ± 2.91	5.70^b^ ± 1.04	3.08^a^ ± 0.40	25.91^a^ ± 2.50
10,000 ppm	57.38^a^ ± 2.56	33.13^a^ ± 4.10	5.75^a^ ± 0.79	24.25^a^ ± 2.24	27.38^a^ ± 3.67	4.34^a^ ± 0.31	5.90^b^ ± 0.87	2.53^a^ ± 0.62	23.71^a^ ± 3.12
15,000 ppm	58.80^a^ ± 0.57	34.73^a^ ± 0.38	6.27^a^ ± 0.66	24.07^a^ ± 2.94	28.46^a^ ± 2.28	4.84^a^ ± 1.25	6.25^b^ ± 0.49	4.28^a^ ± 1.40	28.79^a^ ± 4.79
Stalk fiber fraction and nutrient composition									
Control	57.08^a^ ± 4.76	39.85^a^ ± 4.96	6.06^a^ ± 0.92	15.41^a^ ± 4.82	32.56^a^ ± 2.16	5.66^a^ ± 0.38	3.67^a^ ± 0.95	2.08^a^ ± 0.64	24.45^b^ ± 1.49
5000 ppm	59.12^a^ ± 5.94	38.07^a^ ± 2.23	6.63^a^ ± 0.53	22.86^a^ ± 1.77	32.66^a^ ± 1.31	5.39^a^ ± 0.45	2.50^a^ ± 0.27	2.54^a^ ± 0.49	26.66^ab^ ± 0.50
10,000 ppm	58.77^a^ ± 2.49	36.99^a^ ± 1.56	6.42^a^ ± 0.72	21.78^a^ ± 1.36	30.57^a^ ± 0.85	5.43^a^ ± 0.51	2.73^a^ ± 0.31	1.63^a^ ± 0.75	27.55^a^ ± 0.89
15,000 ppm	59.13^a^ ± 2.20	36.93^a^ ± 1.65	6.44^a^ ± 0.67	22.20^a^ ± 1.55	30.50^a^ ± 0.98	5.65^a^ ± 1.01	3.40^a^ ± 1.69	1.98^a^ ± 0.60	26.36^ab^ ± 0.95

Data is expressed as mean ± SD (*n* = 3). Different lower superscript letters within each column indicate a significant difference within treatments (*p* < 0.05)

*NDF* neutral detergent fiber, *ADF* acid detergent fiber, *ADL* acid detergent lignin, *HEM* hemicellulose, *CEL* cellulose, *LIG* lignin, *CP* crude protein

### In vitro digestibility of leaves and stalks

Results on the in vitro digestibility of leaves and stalks are presented in Table 7. No significant differences were noted in the in vitro true digestibility of dry matter (IVTD), digestible organic matter (DOM), metabolic energy (ME), short-chain fatty acids (SCFA), total digestible nutrients (TDN), net energy (NE), and microbial protein (MP) in the leaves and stalks among all salinity treatments and control.

**Table 7:** Results of in vitro digestibility of leaves and stalks of *Sorghum bicolor* cultivated under different salinity treatments

Treatment	IVTD (DM %)	DOM (%)	ME (MJ kg^−1^ DM)	ME (Mcal kg^−1^ DM)	SCFA (mmol ml^−1^ gas)	TDN (%)	NE (Mcal IB^−1^)	MP (g kg^−1^ DOM)
Leaves in vitro digestibility								
Control	41.50^a^ ± 9.06	49.93^a^ ± 4.92	7.28^a^ ± 0.72	1.74^a^ ± 0.17	0.72^a^ ± 0.13	49.17^a^ ± 3.86	3.68^a^ ± 0.14	59.01^a^ ± 5.92
5000 ppm	33.46^a^ ± 9.16	46.44^a^ ± 2.43	6.95^a^ ± 0.36	1.66^a^ ± 0.09	0.70^a^ ± 0.05	47.40^a^ ± 1.91	3.43^a^ ± 0.12	56.01^a^ ± 2.93
10,000 ppm	29.54^a^ ± 8.01	43.74^a^ ± 3.34	6.52^a^ ± 0.52	1.56^a^ ± 0.12	0.63^a^ ± 0.09	45.11^a^ ± 2.76	3.35^a^ ± 0.12	52.76^a^ ± 4.03
15,000 ppm	33.71^a^ ± 7.51	50.09^a^ ± 5.66	7.48^a^ ± 1.63	1.79^a^ ± 0.39	0.79^a^ ± 0.28	50.26^a^ ± 4.74	3.57^a^ ± 0.36	60.42^a^ ± 6.87
Stalks in vitro digestibility								
Control	33.96^a^ ± 5.17	45.85^a^ ± 4.34	6.88^a^ ± 0.65	1.64^a^ ± 0.16	0.72^a^ ± 0.11	47.02^a^ ± 3.48	3.31^a^ ± 0.14	55.31^a^ ± 5.24
5000 ppm	32.07^a^ ± 2.53	45.33^a^ ± 1.37	6.82^a^ ± 0.23	1.63^a^ ± 0.05	0.71^a^ ± 0.04	46.74^a^ ± 1.20	3.25^a^ ± 0.06	54.68^a^ ± 1.65
10,000 ppm	34.68^a^ ± 5.53	49.01^a^ ± 4.11	7.37^a^ ± 0.64	1.77^a^ ± 0.15	0.81^a^ ± 0.11	49.70^a^ ± 3.44	3.36^a^ ± 0.13	59.12^a^ ± 4.95
15,000 ppm	32.89^a^ ± 5.55	44.83^a^ ± 2.82	6.72^a^ ± 0.10	1.61^a^ ± 0.02	0.69^a^ ± 0.00	46.18^a^ ± 1.56	3.27^a^ ± 0.09	54.08^a^ ± 1.99

Data is expressed as mean ± SD (*n* = 3). Different lower superscript letters within each column indicate a significant difference within treatments (*p* < 0.05)

*IVTD *in vitro true digestibility of dry matter, *DOM* digestible organic matter, *ME* metabolic energy, *SCFA* short-chain fatty acids, *TDN* total digestible nutrients, *NE* net energy, *MP* microbial protein

### Correlation analysis between fiber fraction, nutrient composition, and in vitro digestibility of leaves and stalks

**Fig. 5:**
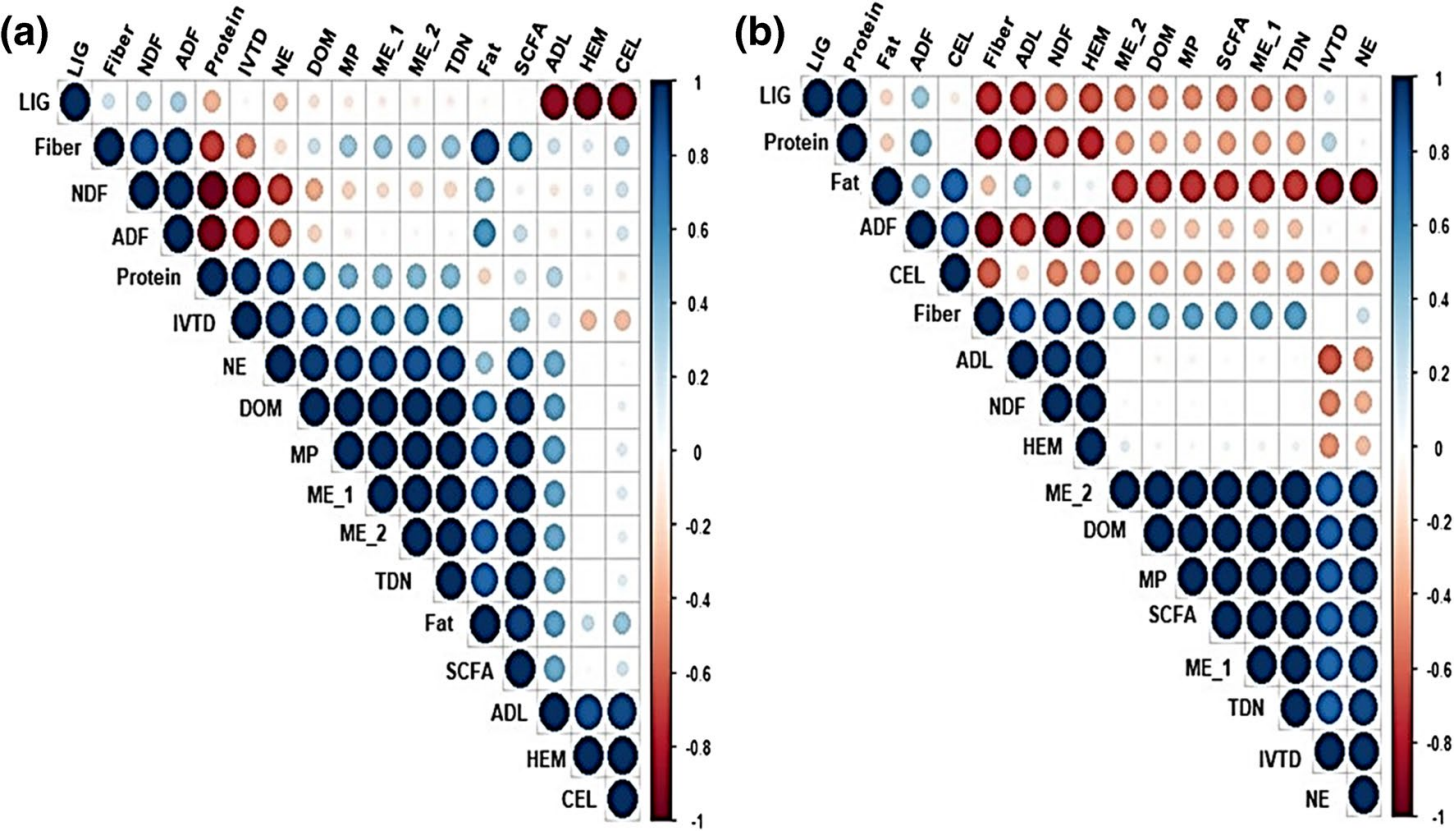
A correlogram showing correlation analysis of fiber fraction, nutrient composition, and in vitro digestibility in (**a)** leaves and (**b)** stalks of sorghum. Blue and brown colors are positive and negative significant correlations, respectively, according to Pearson’s correlation analysis. The color intensity and circle size are proportional to the correlation coefficient. LIG, lignin; HEM, hemicellulose; CEL, cellulose; NDF, neutral detergent fiber; ADF, acid detergent fiber; ADL, acid detergent lignin; IVTD, in vitro true digestible dry matter; DOM, digestible organic matter; ME, metabolic energy; SCFA, short-chain fatty acids; NE, net energy; TDN, total digestible nutrients

Figure 5 shows results of the Pearson’s correlation analysis between fiber fraction, nutrient composition, and in vitro digestibility of leaves and stalks of sorghum cultivated under different treatments in the IAAS. For the leaves (Fig. 5a), there was a significantly strong and positive correlation between NDF and ADF. Likewise, there was a strong and positive correlation between ADL, HEM, and CEL. A significantly strong and positive correlation was also noted between the fiber content and NDF, ADF, ADF, and fat content. IVTD was positively correlated with NE, DOM, MP, ME, and TDN. For stalks (Fig. 5b), there was a significant and positive correlation between ADF and CEL. Likewise, a significant positive correlation was noted between ADL, NDF, and HEM. The fiber content strongly and significantly correlated with ADL, NDF, and HEM. ME strongly and significantly correlated with DOM, MP, SCFA, TDN, and NE.

### Fish growth performance

Table 8 summarizes the results of the growth performance of *Pangasianodon hypophthalmus* reared under different salinity treatments. No significant differences in the final weight were observed among all the salinity treatments. However, data on the body weight gain (BWG) indicated that 15,000 ppm salinity treatment significantly recorded lower values for the BWG compared to 10,000 ppm and 5000 ppm salinity treatments (*p* < 0.05). Likewise, the 15,000 ppm treatment significantly (*p* < 0.05) recorded the highest values for the feed conversion ratio (FCR) compared to 10,000 ppm and 5000 ppm salinity treatments. Data on the specific growth rate (SGR) indicated that fish reared under the 5000 and 10,000 ppm salinity treatments significantly (*p* < 0.05) had higher SGR compared to the 15,000 ppm treatment (*p* < 0.05). For the condition factor (CF), fish reared under 5000 ppm significantly (*p* < 0.05) recorded higher values for CF compared to those reared under 15,000 ppm treatment. No significant differences in the survival rate were noted among all the salinity treatments.

**Table 8:** Growth performance parameters of *Pangasianodon hypophthalmus reared under different salinity treatments*

Growth performance	5000 ppm	10,000 ppm	15,000 ppm
Initial weight (g)	6.00^a^ ± 0.00	6.00^a^ ± 0.00	6.00^a^ ± 0.00
Final weight (g)	111.26^a^ ± 7.56	111.70^a^ ± 8.32	63.40^b^ ± 3.72
BWG (g)	105.26^a^ ± 7.56	105.70^a^ ± 8.32	57.40^b^ ± 3.72
FI (g)	108.98	109.21	124.02
FCR	1.04^b^ ± 0.07	1.04^b^ ± 0.08	2.17^a^ ± 0.14
SGR (%)	1.65^a^ ± 0.04	1.65^a^ ± 0.04	1.33^b^ ± 0.03
CF	1.09^a^ ± 0.19	0.91^ab^ ± 0.25	0.87^b^ ± 0.12
Survival (%)	89.00^a^ ± 0.00	91.00^a^ ± 0.00	70.00^a^ ± 0.00

Data is expressed as mean ± SD (*n* = 3). Different lower superscript letters within each row indicate a significant difference within treatments (*p* < 0.05)

*BWG* body weight gain, *FI* feed intake, *FCR* feed conversion ratio, *SGR* specific growth rate, *CF* condition factor

### Hematological and serum biochemical parameters

**Fig. 6:**
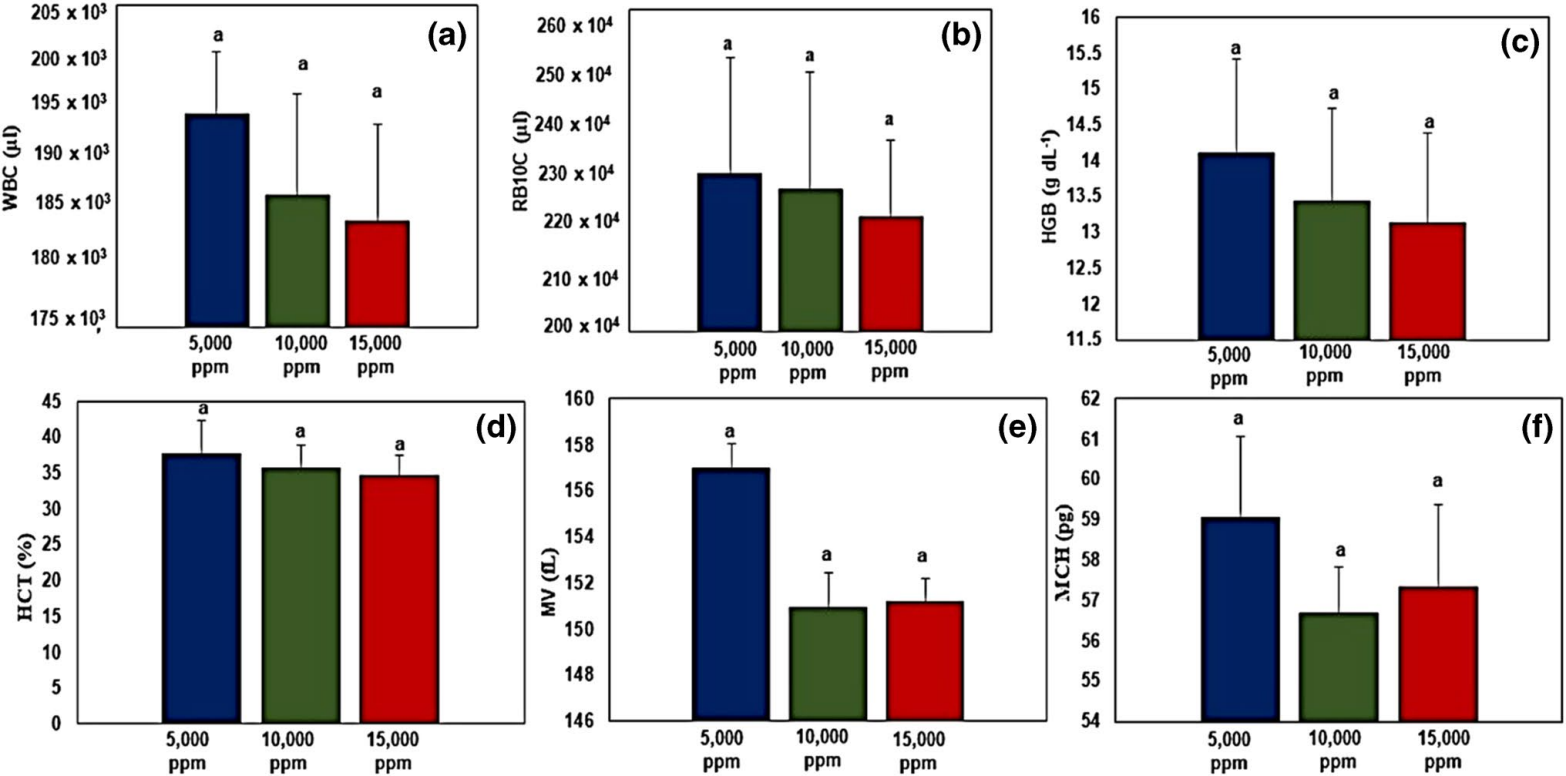
Hematological parameters of fish reared under different salinity treatments. **a** White blood cells (WBC), **b** red blood cells (RBC), **c** hemoglobin (HGB), **d** hematocrit (HCT), **e** mean corpuscular volume (MCV), **f** mean corpuscular hemoglobin (MCH). Data presented as mean ± SD (*n* = 3). Error bars represent standard deviation. Bar columns having different letters are significantly different (*p* < 0.05)

The results of the hematological parameters are presented in Fig. 6. No significant differences in the concentration of white blood cells (Fig. 6a), red blood cells (Fig. 6b), hemoglobin (Fig. 6c), hematocrit (Fig. 6d), mean corpuscular volume (Fig. 6e), and mean corpuscular hemoglobin (Fig. 6f) were noted across all salinity treatments.

**Fig. 7:**
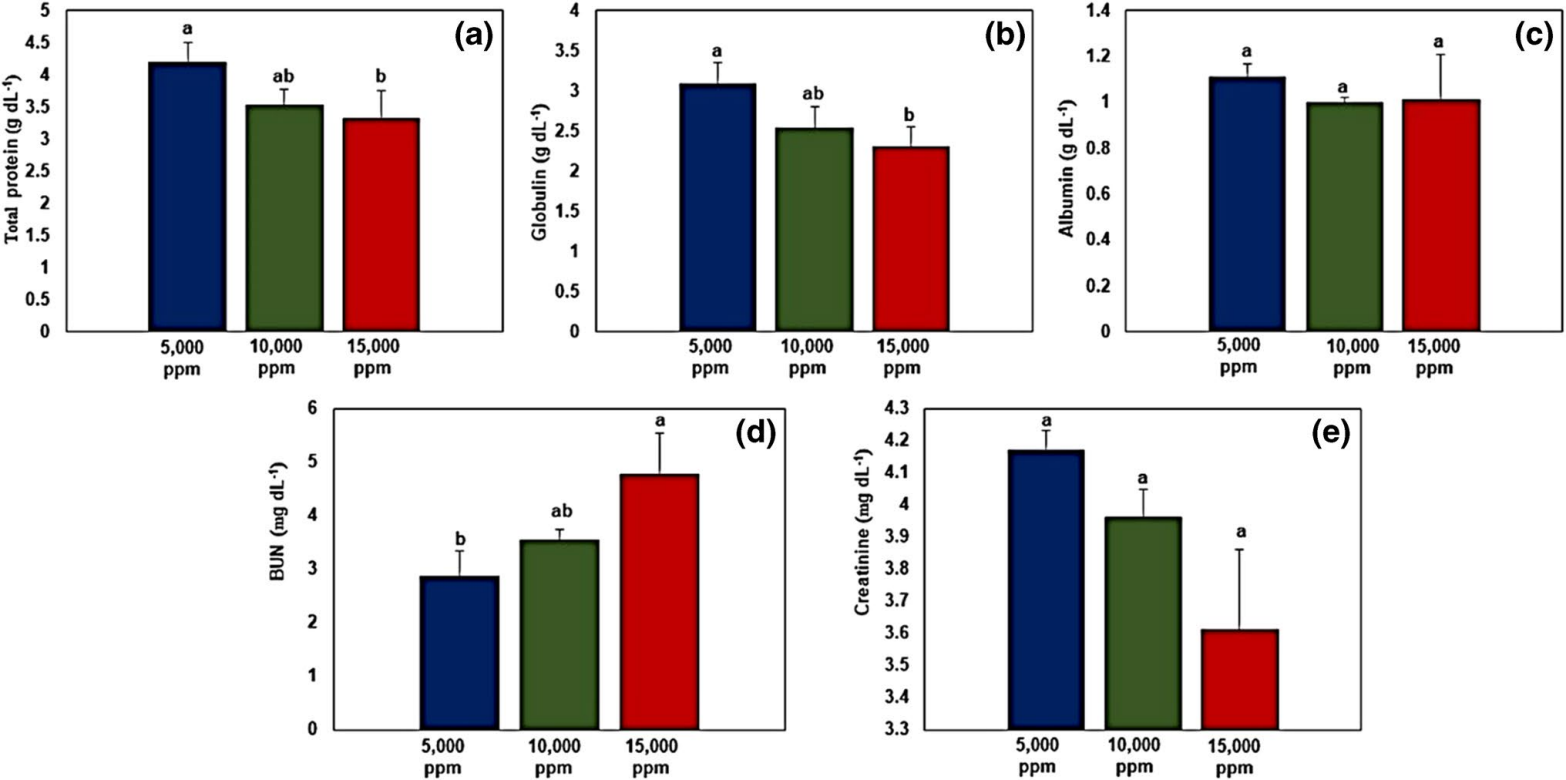
Serum biochemical parameters of fish reared under different salinity treatments. **a** Total protein, **b** albumin, **c** globulin, **d** blood urea nitrogen (BUN), and **e** creatinine. Data presented as mean ± SD (*n* = 3). Error bars represent standard deviation. Bar columns having different letters are significantly different (*p* < 0.05)

Data on the serum biochemical parameters are presented in Fig. 7. Results indicated that 5000 ppm salinity treatment significantly (*p* < 0.05) recorded higher values for serum total protein concentration compared to 15,000 ppm treatment (Fig. 7a). No significant differences in albumin concentration were noted among all the salinity treatments (Fig. 7b). However, the 5000 ppm salinity treatment significantly (*p* < 0.05) recorded higher values for globulin concentration compared to the 15,000 ppm treatment (Fig. 7c). For blood urea nitrogen (BUN), the results showed that 15,000 ppm salinity treatment significantly (*p* < 0.05) recorded higher values for BUN compared to 10,000 ppm treatment (Fig. 7d). No significant differences in creatinine concentration were noted among all salinity treatments (Fig. 7e).

### Fish wastewater quality

**Fig. 8:**
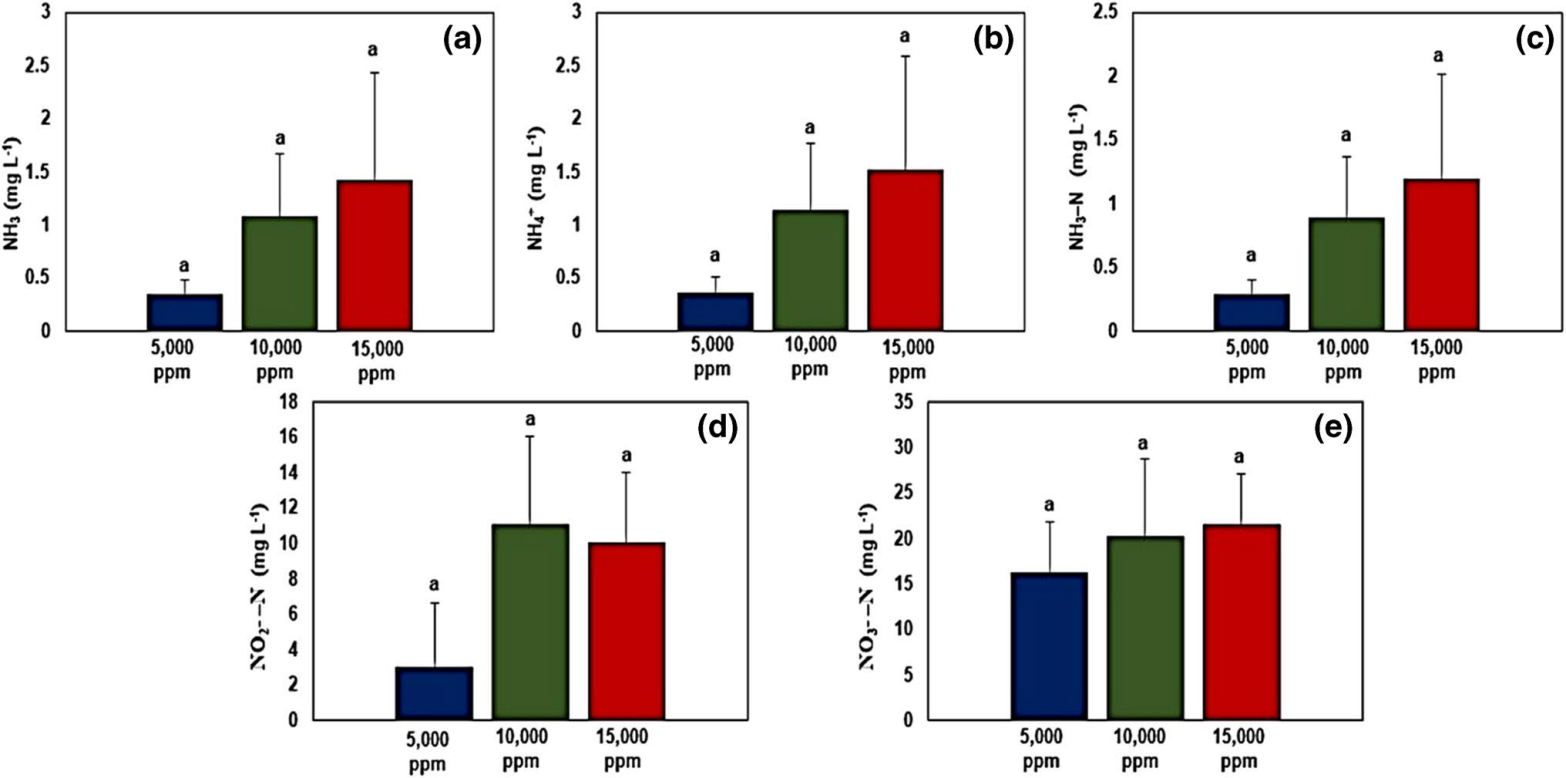
Water quality parameters of fish wastewater under different salinity treatments. **a** NH_3_ (ammonia); **b** NH_4_^+^ (ammonium); **c** NH_3_–N (ammonia–nitrogen); **d** NO_2_^ −^ –N (nitrite–nitrogen); **e** NO_3_^ −^ –N (nitrate–nitrogen). Data expressed as mean ± SE. Error bars represent the standard error. Different lower superscript letters within each column indicate a significant difference within treatments (*p* < 0.05)

The fish wastewater quality parameters are presented in Fig. 8. Although the 5000-ppm salinity treatment recorded the lowest values for ammonia, ammonium, ammonia–nitrogen, nitrite–nitrogen, and nitrate nitrogen, no significant differences were noted with other salinity treatments.

## Discussion

The reuse of aquaculture wastewater in agriculture is becoming more prominent in arid and semi-arid regions of the world due to water scarcity (Gengmao et al. 2010; El-kady and Suloma 2013; Chen et al. 2017; Calone et al. 2020). Aquaculture wastewater is rich in organic matter and nutrients such as nitrogen (N), phosphorus (P), and potassium (K) which are important for plant growth (Omotade et al. 2019; Kimera et al. 2023; Terkula et al. 2023). In our study, irrigating sorghum with saline aquaculture wastewater did not show significant detrimental effects in the tested agro-morphological parameters (plant height, leaf area, stalk diameter, and SPAD) and hence in agreement with previous studies (Guimarães et al. 2019, 2022; Kolozsvári et al. 2022). This is because sorghum is moderately tolerant to soil and water salinity of up to 5440 and 2700 ppm respectively above which a 16% reduction in yield is expected (Calone et al. 2020). For the number of internodes per plant, it was observed that the three salinity treatments significantly decreased the internode number compared to the control. This is because the accumulation of sodium ions in plant tissues is toxic, and it affects cell division and expansion (Alizadeh et al. 2011). On the other hand, however, higher water salinities (i.e., 5000 ppm, 10,000 ppm, and 15,000 ppm) did not show significant differences in both forage and grain yield with the control treatment. This could be attributed to several factors such as the presence of plant growth-promoting bacteria (PGPBs) in aquaculture wastewater and the crop variety cultivated. Previous literature indicates that PGPBs not only promote plant growth but also act as elicitors of salinity tolerance in plants (Vacheron et al. 2013; Daliakopoulos et al. 2016; Kumar et al. 2020). Furthermore, an increase in hemicellulose content in leaves and stalks of sorghum was noted especially under highly saline conditions (10,000 ppm and 15,000 ppm) which we anticipate could have led to improved salinity tolerance in sorghum. Tissue-specific up- and down-regulations of the expression of cell wall remodeling enzymes such as xyloglucan endotransglucosylase/hydrolases (XTHs) responsible for hemicellulose biosynthesis have previously been linked to positively or negatively regulate salinity tolerance in several plant species such as *Salicornia europaea* (Tiika et al. 2021), *Medicago truncatula* (Xuan et al. 2016), *Vitis vinifera* L. (Qiao et al. 2022), and *Populus* sp. (Cheng et al. 2021).

Pearson’s correlation coefficient analysis indicated positive correlations between SPAD (leaf chlorophyll content) and yield parameters (i.e., grain yield, fresh and dry forage yield). Chlorophyll has a direct role in photosynthesis and thus has an impact on the photosynthetic capacity, growth, and yield of crops (Liu et al. 2019; Song et al. 2019; Wasaya et al. 2021; Zhou et al. 2022). In our study, lower forage and grain yields, although non-significant, were noted in highly saline treatment conditions compared to the control, and this correlated with a decrease in SPAD values. Salinity not only disintegrates the chlorophyll structure and integrity but also denatures enzymes responsible for chlorophyll biosynthesis, hence leading to a decrease in chlorophyll content of leaves, growth, and yield (Mugwanya et al. 2022; Nawaz et al. 2022). Similar results have previously been reported in *Oryza sativa* L. (Hakim et al. 2014; Kumar et al. 2021), *Triticum aestivum* (Eroglu et al. 2020; Adil et al. 2022), *Hordeum vulgare* L. (Noreen et al. 2021; Alharbi et al. 2022), and *Zea mays* (Bouras et al. 2021; Ali et al. 2022).

The current study also indicated variations in seed macro- and micronutrient contents in response to different salinity treatments and the control. High accumulation of micronutrients such as zinc (Zn), copper (Cu), manganese (Mn), and iron (Fe) in seeds of sorghum plants cultivated under high salinity (15,000 ppm) is in agreement with previous studies on *Triticum aestivum* (Faran et al. 2019), *Cucumis sativus* L. (Mugwanya et al. 2022), *Hordeum vulgare* L. (Ali et al. 2012), and *Gossypium hirsutum* L. (Dai et al. 2014). Micronutrients such as Zn, Cu, and Fe have been linked to play a significant role in antioxidant enzyme activity under plant abiotic stress (Bian and Jiang 2009; Babaei et al. 2017; Hassan et al. 2020). In the same regard, increased accumulation of calcium (Ca) in seeds of sorghum plants cultivated under high salinity (15,000 ppm) indicates the role of Ca in modulating abiotic stress. For instance, calcium-dependent protein kinases (CDPKs) have been reported to modulate abiotic stress through activation and regulation of several enzymes, genes, transcription factors, and ion channels, hence supporting plant adaptation to different abiotic stress factors (Franz et al. 2011; Atif et al. 2019; Aslam et al. 2021).

The forage quality in terms of fiber fraction and nutrient composition of the sorghum stover (leaves and stalks) was assessed. Generally, stalks had higher NDF, ADF, and ADL contents compared to the leaves and this is in agreement with previous studies (Kaplan et al. 2014; Lamidi and Konyeha 2020; Wahyono et al. 2021). Moreover, the fiber content in stalks was higher in plants irrigated with saline aquaculture wastewater compared to the control. Fiber is majorly composed of cellulose and lignin (Yang et al. 2019). However, it has been previously reported that the lignification of plant tissues increases with an increase in salinity stress, but this is species-dependent (Zou et al. 2021; Wang et al. 2023). Except for the control treatment, the crude protein (CP) content of leaves and stalks of sorghum was below the recommended range (7%) for good-quality forage (Milford and Minson 1966) in all the salinity treatments. This could be attributed to a higher concentration of N in the chemical fertilizers used in the control treatment relative to that in saline aquaculture wastewater. Previous studies have also shown that the application of N fertilizers in sorghum improves their forage quality in terms of crude protein content (Ates et al. 2019; Holman et al. 2019; Sriagtula et al. 2019). Rumen fermentation represented as in vitro true digestible dry matter (IVTD), digestible organic matter (DOM), metabolic energy (ME), net energy (NE), and short-chain fatty acids (SCFA) was assessed in both leaves and stalks. Lower values for IVTD, DOM, ME, NE, and SCFA were recorded in contrast to recent studies on brown midrib mutant (BMR) sorghum (Wahyono et al. 2019, 2023; Umesh et al. 2022) and BMR millet (Oskey et al. 2023). The difference in results could be attributed to lower lignin and fiber contents in BMR forage crops compared to non-BMR forage and grain crops. This is because lignin, a complex phenolic polymer deposited in the secondary cell wall of plants, slows down the enzymatic breakdown of complex sugars such as cellulose, hence decreasing the forage digestibility in ruminants (Saluja et al. 2021). Moreover, a meta-analysis of dairy cows fed conventional sorghum or corn silages compared with BMR silage sorghum indicated improved milk production, increased milk fat concentration, and lactose yield in cows fed on BMR silage sorghum compared to those fed conventional sorghum or corn silage (Sánchez-Duarte et al. 2019).

Stripped catfish (*Pangasianodon hypophthalmus*) were reared under different water salinities (i.e., 5000 ppm, 10,000 ppm, and 15,000 ppm), and results indicated lower values for final weight, body weight gain (BWG), and specific growth rate (SGR) in fish reared at 15,000 ppm compared to those reared at 5000 and 10,000 ppm. This could be attributed to the lower feed intake of fish due to salinity stress which resulted in poor feed conversion ratio (FCR) and hence in agreement with previous studies (Nguyen et al. 2014; Meritha et al. 2018; Ha et al. 2021; Hieu et al. 2021). *P. hypophthalmus* is a freshwater fish species whose osmoregulation is disrupted when exposed to water salinity levels exceeding 10,000 ppm (Nguyen et al. 2014). Moreover, the disruption of osmoregulation negatively impacted the health status of fish as indicated by a decline in values for hematological parameters (red blood cells, white blood cells, hemoglobin, and hematocrit; Fig. 6), serum biochemical parameters (total protein, globulin, and creatinine; Fig. 7), and water quality (i.e., accumulation of nitrogenous compounds in water; Fig. 8).

## Conclusion

According to our study’s findings, irrigating grain sorghum with aquaculture wastewater of varying salinity levels (i.e., 5000 ppm, 10,000 ppm, and 15,000 ppm) leads to the accumulation of toxic ions in plant tissues which negatively impacts certain plant growth parameters most notably the internode number. However, these salinity treatments do not lead to significant reductions in forage and grain yield production. Likewise, these salinity levels do not significantly reduce the nutritive composition of sorghum seeds as well as the forage quality of leaves and stalks in terms of fiber fraction, nutritive composition, and in vitro digestibility. Although rearing striped catfish (*Pangasianodon hypophthalmus*) at salinity levels above 10,000 ppm negatively impacts its growth performance, this fish species can still survive at water salinities reaching up to 15,000 ppm. Overall, the integration of sorghum production and striped catfish production under salinity levels of 5000 ppm could enhance economic returns to farmers regarding increased grain, forage, and fish yield in conditions of freshwater scarcity.

## Data Availability

The datasets generated during and/or analyzed during the current study are available from the corresponding author on reasonable request.

## Declarations

### Plant material

All plant materials and related procedures in this study were done in accordance with the guidelines of the Institutional Review Board of the American University in Cairo and the Ministry of Agriculture and Land Reclamation in Egypt.
